# Nijmegen breakage syndrome and chronic polyarthritis

**DOI:** 10.1186/1824-7288-39-59

**Published:** 2013-09-17

**Authors:** Srdjan Pasic, Maja Cupic, Tanja Jovanovic, Slobodanka Djukic, Maja Kavaric, Ivana Lazarevic

**Affiliations:** 1 Pediatric Immunology, Mother and Child Health Institute, Medical Faculty, University of Belgrade, 8 R. Dakica Street, Belgrade 11070, Serbia; 2 Institute for Microbiology and Immunology, Medical Faculty, University of Belgrade, Belgrade, Serbia; 3 Department of Hematology, Institute for diseases in childhood Clinical center of Montenegro, Podgorica, Montenegro

**Keywords:** Nijmegen breakage syndrome, Juvenile idiopathic arthritis, Polyarthritis

## Abstract

We report on pediatric patient with Nijmegen breakage syndrome (NBS), a rare DNA repair disorder characterized by microcephaly, immunodeficiency and predisposition to malignant lymphomas, who developed juvenile idiopathic arthritis (JIA)-like polyarthritis. In patients with primary immunodeficiencies (PID), septic arthritis due to pyogenic bacteria or mycoplasmal arthritis are the most common osteoarticular manifestations. In certain PID, chronic, non-infectious arthritis resembling rheumatoid arthritis may occur. In our patient microbiologic cultures of synovial fluid including *Mycoplasma spp*. were negative. At first, because of suspected mycoplasmal arthritis we used macrolides and doxycycline combined with hydroxychloroquine but without therapeutic response. However, the use of rituximab led to remission of her polyarthritis lasting for 9 months. Autoimmune features were rarely reported in NBS. An occurrence of JIA-like, chronic polyarthritis in NBS, a DNA repair disorder characterized by decreased tolerance of immunosuppressive drugs such as methotrexate and a high natural risk for lymphomas, makes therapeutic approach even more complex.

## Introduction

Nijmegen breakage syndrome (NBS) is a rare autosomal recessive DNA repair disorder characterized by microcephaly, immunodeficiency and cancer [1,2]. NBS is due to hypomorphic mutations of *NBS1* gene, encoding less functional nibrin, a protein involved in the repair of DNA double-strand breaks and in cell cycle checkpoints [3]. Albeit NBS has been reported in different ethnic groups, more than 90% of affected persons are of Slavic origin (Central-East Europe) and they carry homozygous founder mutation, a 5- base-pair deletion 657del5 [4].

In NBS, congenital osteoarticular malformations such as clinodactyly, polydactyly or syndactyly are the most common occuring in one-half of the patients. Hydronephrosis, hypoplastic kidney, anal atresia/stenosis, CNS malformations or gonadal failure are less commonly observed [2,4].

In several other primary immunodeficiencies (PID) non-infectious, chronic polyarthritis resembling rheumatoid arthritis (RA) or juvenile idiopathic arthritis (JIA) has been reported [5-7].

We report a JIA-like, chronic polyarthritis in a female patient with NBS.

## Case report

This 12-year-old girl of Slavic origin is the first child of healthy, unrelated parents. During neonatal period intrauterine growth retardation (birth weight, 2700 grams) and microcephaly (head circumference, HC = 31 cm; < p3) were observed. Her past medical history revealed that she suffered from recurrent lung infections (otitis media, pneumonia) since infancy. At 18 months of age she was treated for bacterial meningitis. At 8 years of age she was hospitalized because of severe varicella complicated with pneumonia. At the same age she had an episode of hip pain diagnosed as transitory synovitis.

**Figure 1:**
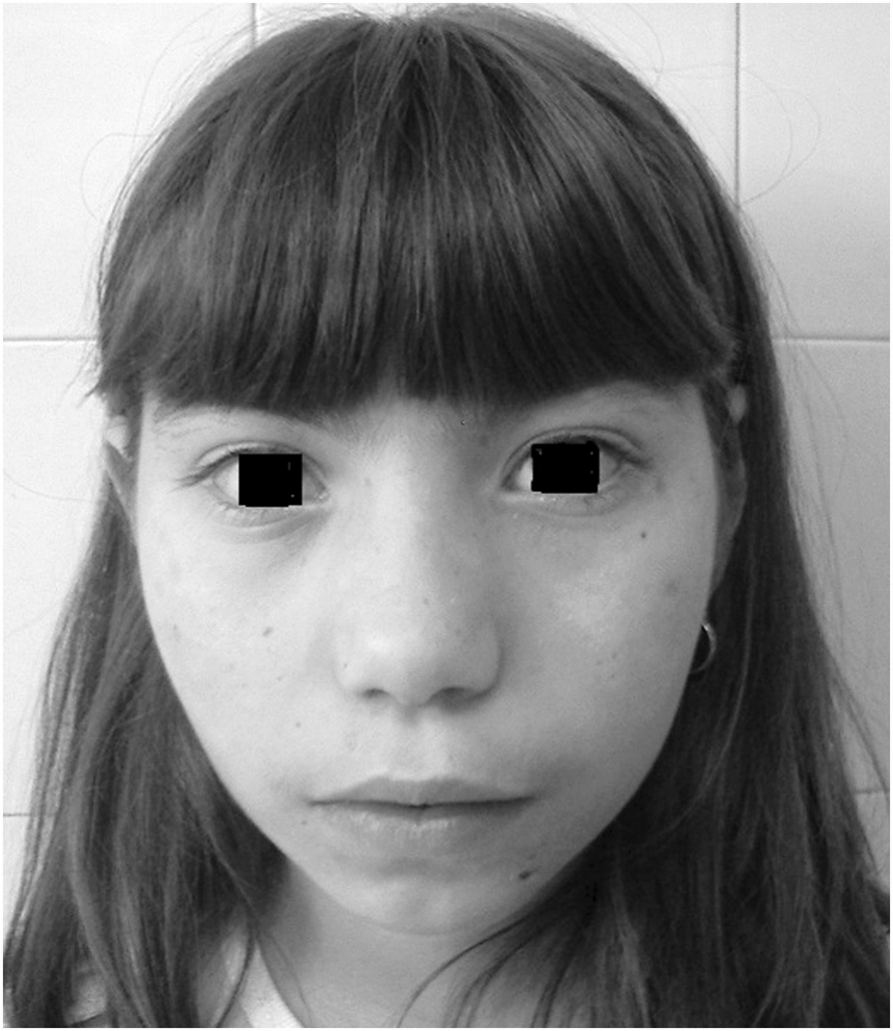
Microcephaly and typical bird-like appearance in our patient.

At 9 years of age she was referred to our Institute for investigation of repeated lung infections. Physical examination on admission included: height, 129 cm (p50) and the body weight, 29 kg (p50); microcephaly (head circumference = 46 cm, < p3); typical facial features with prominent midface, large ears and hyperthelorism with upward slant of the palpebral fissures (Figure 1); hypopigmented spots on trunk and extremities; bilateral clinodactyly and syndactyly of the second and third toe were observed. Chest examination revealed bilateral basilar crackles due to bronchiectasis. Neurological examination and ophtalmologic slit-lamp examination were normal.

Immunologic investigations are presented in Table 1. Karyotype of peripheral blood lymphocytes revealed typical chromosomal rearrangements involving chromosomes 7 and 14. Final diagnosis of NBS was established by mutation analysis of the *NBS1* gene that revealed homozygousity for typical 5 base-pair deletion (657del5). She was placed on regular substitution with intravenous immunoglobulin (IVIG) in a dose of 400 milligrams/kg/body weight every 4 weeks. With this treatment acute exacerbations of her chronic lung disease decreased in frequency.

**Table 1:** Immunologic investigations in our patent

	**At diagnosis**	**At the onset of arthritis**	**Normal values for given age**
IgA (g/l)	0.08	0.07	0.11-2.51
IgM	0.04	0.06	0.13-2.51
IgG	0.70	10.8^b^	6.54-15.94
IgE (IU/ml)	< 50	-	
Absolute lymphocyte count (mm^3^)	1512	1340	1100-5900
CD3+ lymphocytes	726	650	700-4200
CD4+	333	252	300-2000
CD8+	348	396	300-1800
CD19+	36	42	200-1600
CD3-CD16 + C56+ (NK-cells^a^)	665	720	9-900
Proliferative lymphocyte response using phytohemagglutinin (PHA)	Pt. SI^c^ 18×	-	-
	Control SI 87×		

^a^ NK-cells – natural killer; ^b^ IgG trough level during replacement therapy with IVIG; ^c^*SI* stimulation index.

**Figure 2:**
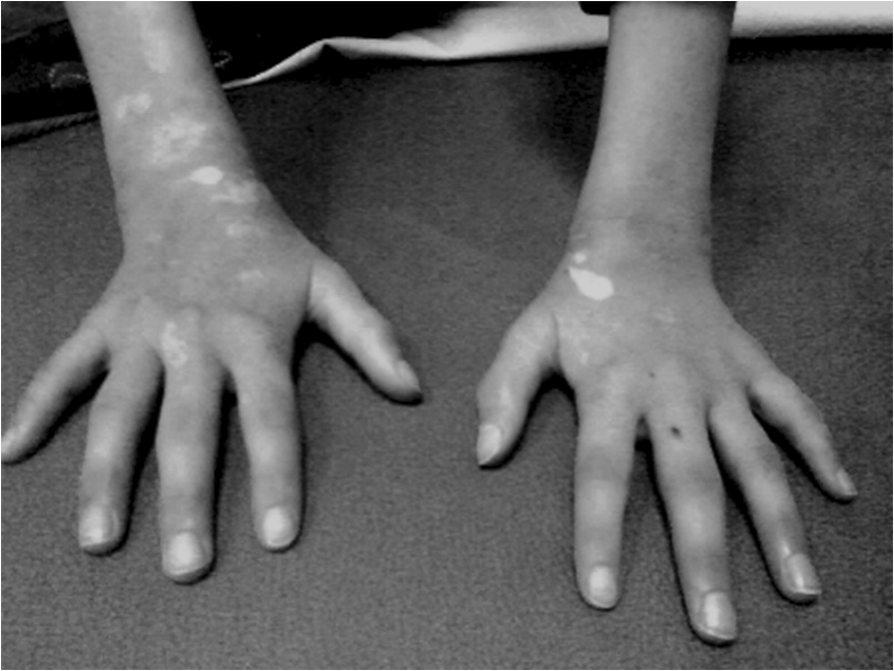
Symmetrical arthritis of proximal interphalangeal, metacarpophalangeal and radiocarpal joints (also, note vitiligo).

At 10 years of age she was admitted because of pain, morning stiffness and bilateral swelling of her proxymal interphalangeal (PIP), wrist and knee joints (Figure 2). Affected joints were hot, swollen and tender on passive or active motion. L Laboratory investigations at that time included: erythrocyte sedimentation rate 42 mm/hr, C-reactive protein 92 mg/l (normal < 5 mg/l), hemoglobin 121 g/l, WBC count 6.2 × 10^9^/l with 58% of neutrophils, 30% of lymphocytes and 6% of monocytes, platelets were 346 × 10^9^/l; urinalysis was normal; liver function tests was normal; serum lactate-dehydrogenase was not increased; serum concentrations of complement components C3, 1.99 g/l and C4, 0.46 g/l both were increased. Immunologic investigations including serum immunoglobulin concentrations, peripheral blood lymphocyte phenotype and proliferative response of lymphocytes to mitogen are presented in Table 1. Antinuclear antibodies, anti-double stranded DNA, anti-cyclic citrullinated protein antibodies and rheumatoid factor (RF) were all negative.

Diagnostic puncture of knee joint was done and 4 milliliters of cloudy, yellow sinovial exudate was obtained. Microscopic examination and bacterial culture of joint fluid specimen yielded no microogranism. Also, special cultures for *Mycoplasma spp., Ureaplasma urealyticum* and mycobacteria were negative.

An initial treatment included non-steroidal anti-inflammatory drug (NSAID) meloxicam together with azithromycin because of suspected mycoplasmal infection. After two months of treatment, no improvement was seen so that azithromycin was replaced for doxycycline in a daily dose of 4 mg/kg/body weight and with this treatment her joint symptoms transiently improved. However, over the next 6 months several relapses of arthritis were observed so hydroxychloroquine as disease-modifying antirheumatic drug (DMARD) was given.

After one year of treatment no objective improvement of polyarthritis using defined core set of six variables for JIA (physician global assessment of disease activity; parent global assessment of disease activity; number of joints with active arthritis; number of joints with limited range of motion; child health assessment questionaiire, erythrocyte sedimentation rate) was observed [8,9].

We decided to introduce rituximab in a standard dose regimen (375 mg/m^2^/month) used in children and adults affected with PID who developed autoimmune diseases [10]. She received six infusions of rituximab without major side-effects and continued to receive regular IVIG therapy. Treatment with rituximab led to complete clinical and laboratory remission of her polyarthritis lasting for 9 months (Table 2).

**Table 2:** Assessment of disease activity using six defined variables

**Assessment of disease activity**	**Before rituximab**	**After rituximab**
Physician VAS	68	10
Parental VAS	73	8
N of joints with active arthritis	15	0
N of joints with limited range of motion	12	3
CHAQ	2.25	1.25
Erythrocyte sedimentation rate (mm/hr)	80	12

## Discussion

Microcephaly, immunodeficiency and predisposition for lymphoma at an early age are the hallmarks of NBS [2,4]. Other commonly associated features in NBS include skin manifestations such as café-au-lait spots, vitiligo, telangiectasia, flat haemangiomas or sun sensitivity of the eyelids [2,4]. Also, congenital malformations such as clinodactyly or syndactyly of the II/III toes are common occuring in one-half of the patients.

Inflammatory, JIA-like polyarthritis has been reported so far in the single Argentinian patient of Hungarian origin with NBS [11]. Similar to our patient, in Argentinian patient the course of arthritis resembled polyarticular onset JIA, symmetrically affecting small and large joints.

In humoral PID, such as X-linked agammaglobulinaemia (XLA) or common variable immunodeficency (CVID), septic arthritis caused with pyogenic bacteria (*S.aureus, S.pneumoniae, H.influenzae type b*) is the most common osteoarticular manifestation [12]. Infectious arthritis in patients with humoral PID may be also caused with *Mycoplasma spp*. and *U.urealyticum*[12]. Colonisation of mycoplasmal species in these patients is facilitated because of the lack of secretory antibodies at mucosal surfaces, and these organisms easily spread to synovial fluid. Also, patients with XLA or CVID may remain susceptible to mycoplasmal infection even after introduction of regular IVIG therapy because commercial IVIG lots do not contain sufficient titers of specific antibodies against *Mycoplasma spp*.

In patients with PID, differentiating non-infectious arthritis from mycoplasmal infection represents a problem, because culturing of these microorganisms may be difficult. In originally reported patient with NBS and polyarthritis analysis of synovial fluid was not done [11]. Furr *et al.* reported mycoplasmal infection in 8 (38%) of 23 patients with hypogammaglobulinaemia and arthritis [13]. In our patient analysis of synovial fluid showed characteristics of exudate but microbiologic investigations was negative. At first, we treated our patient with macrolides and doxycycline but without therapeutic response.

In a subset of patients with NBS, similar to CVID, immunodeficiency is characterized with lymphopenia associated with a state of “immune dysregulation” leading to autoimmunity [14]. Opposite to CVID, autoimmune disorders such as autoimmune hemolytic anemia, thrombocytopenia or polyarthritis were rarely reported in NBS [4,11]. In approximately 20% of patients with CVID, a spectrum of autoimmune disorders can arise, with autoimmune cytopenias being the most common, followed with RA, pernicious anemia, primary biliary cirrhosis, thyroiditis, sicca syndrome, systemic lupus or inflammatory bowel disease, respectively [15]. In a subset of patients with granulomatous form of CVID characterized with lymphopenia, autoimmune disorders, including rheumatoid-like arthritis, were even more common, occurring in more than one-half of patients [7]. In CVID, low proportions of CD4 + CD25+, Foxp3 T regulatory cell subset correlated with presence of autoimmunity and splenomegaly [16]. In patients with NBS regulatory T cells so far were not investigated.

In our patient the use of NSAID and hydroxychloroquine led to short-term improvement of arthritis. Hydroxychloroquine was the most commonly used in patients with CVID who developed rheumatoid-like arthritis or systemic lupus [7]. In a few patients with PID presenting with non-infectious arthritis an introduction of regular IVIG therapy was effective controlling articular symptoms [12]. By contrast, our and originally reported patient both experienced relapses of severe polyarthritis while receiving regular treatment with IVIG [11].

In CVID, cautious use of steroids has been employed for treatment of various autoimmune features including arthritis [6,7]. Rosenzweig *et al.* also observed an initial improvement of JIA-like arthritis in NBS patient using prednisone [11]. We were reluctant to commence steroids because of the risk for severe infections. The use of low-dose methotrexate, a DMARD with well established safety and efficacy in children with JIA, was considered in Argentinian patient with NBS [11]. However, previously reported patients with NBS treated for malignancy showed reduced tolerance to methotrexate, alkylating agents, epipodophilotoxines and anthracyclines [17,18]. Therefore, we believe that prolonged use of methotrexate would not be a safe option in NBS patients with arthritis.

Anti-TNF agents are widely accepted treatment for patients with JIA who not respond to treatment with DMARD’s. However, the use of anti-TNF or anti-TNF receptor recombinant fusion monoclonal antibodies (infliximab, etanercept) should be avoided in NBS because of already present high risk for lymphoma.

In our patient an improvement of arthritis was observed after the use of rituximab. Because of profound immunodeficiency we did not use rituximab in a high-dose regimen recommended for treatment of rheumatoid arthritis but instead we employed standard dosage (375 mg/m^2^) that was safely used in PID. In XLA, a condition with total absence of mature B cells, occurence of RA has been rarely reported [19,20]. This observation led to B cell depletion approach with humanized monoclonal antibody rituximab in several rheumatic diseases. In PID, the mechanism of inflammatory, non-infectious arthritis is not well understood. However, in patients with PID and severe, chronic arthritis, depletion of synovial B cells serving as antigen presenting cells to autoreactive T cell clones may be important [21].

## Conclusions

In conclusion, we report on second pediatric patient with NBS who developed chronic, JIA-like polyarthritis. Non-infectious, chronic polyarthritis resembling JIA or RA was the most commonly associated with antibody deficiencies. An occurrence of inflammatory polyarthritis in NBS represents a rare, autoimmune feature in this DNA repair disorder.

## Note added in proof

At the age of 15 years our patient developed T-cell lymphoblastic leukemia/lymphoma (TLBL/ALL) and started treatment according to the protocol (Berlin-Frankfurth-Münster, BFM’09 protocol) for standard risk ALL.

## Consent

Written informed consent was obtained from the parents for the publication of this report and any accompanying images.

## Abbreviations

NBS: Nijmegen breakage syndrome; JIA: Juvenile idiopathic arthritis; RA: Rheumatoid arthritis; PID: Primary immunodeficiency; CVID: Common variable immunodeficiency; IVIG: Intravenous immunoglobulin; DMARD: Disease-modifying antirheumatic drug.

## Competing interests

The authors declare that they have no competing interests.

## Authors’ contribution

SP, MC, IJ, SD, MK and TJ drafted the manuscript. SP make a critical revision. All authors read and approved the final manuscript.
